# A Comprehensive Assessment of Cancer Patient Performance Status Documentation in a Large, Multicentre Hospital System

**DOI:** 10.1111/jep.70411

**Published:** 2026-03-15

**Authors:** Guillaume Lamé, Mohamed El Mejdani, Ariel Cohen, Sonia Priou, Rémi Flicoteaux, Matthew Barclay, Christophe Tournigand, Marie Verdoux, Emmanuelle Kempf

**Affiliations:** ^1^ Laboratoire Génie Industriel, CentraleSupélec Université Paris Saclay Gif‐sur‐Yvette France; ^2^ Innovation and Data, IT Department Assistance Publique—Hôpitaux de Paris Paris France; ^3^ Laboratoire d'Informatique Médicale et d'Ingénierie des Connaissances pour la e‐Santé (LIMICS) Sorbonne University Inserm, Université Sorbonne Paris Nord Paris France; ^4^ European Institute for Innovation through Health Data Ghent Belgium; ^5^ Department of Medical Information Assistance Publique—Hôpitaux de Paris Paris France; ^6^ Epidemiology of Cancer Healthcare and Outcomes, Research Department of Behavioural Science and Health, Institute of Epidemiology and Health Care University College London London UK; ^7^ INSERM U955, Equipe 18 Institut Mondor de Recherche Biomédicale Créteil France; ^8^ Department of Medical Oncology, Henri Mondor and Albert Chenevier University Hospital, Assistance Publique—Hôpitaux de Paris Université Paris Est Créteil Créteil France

**Keywords:** cancer, electronic health records, Karnofsky performance status, routinely collected health data

## Abstract

**Background:**

The performance status (PS) is an indicator of a cancer patient's ability to perform everyday activities and plays a key role in oncology. Research suggests that the documentation of PS scores in electronic health records (EHR) is deficient.

**Methods:**

We analysed PS score documentation (Karnofsky or ECOG/Zubrod/WHO) in the hospital, consultation, and multidisciplinary team meeting (MDT) records of patients newly referred for a cancer at a large, public, multisite hospital system, between 1 January 2019 and 1 June 2021. We developed a regular expression (RegEx) to automatically identify PS in documents and assessed what patient and hospital characteristics were associated with PS documentation.

**Results:**

Our RegEx achieved accuracy, and weighted‐ and macro‐average F1 score, > 0.95 for all document types. We included 68,479 patients. 35% had a documented PS between −90 and +365 days of their first ICD‐10 cancer code. 18% of MDT reports contained a PS score.

In multivariate analysis, without accounting for metastatic status at diagnosis, odds ratios (ORs) for PS documentation in patient files varied by cancer type, from 0.47 (95% confidence interval: [0.42; 0.52]) for genitourinary to 3.30 [3.00; 3.61] for lung cancer, and hospital, from 0.27 [0.23; 0.33] to 3.38 [3.14; 3.63]. Male patients were more likely to have a documented PS (OR = 1.08 [1.04; 1.13]), as well as older patients. The number of each type of document was positively correlated with the presence of a score. When adding metastatic status at diagnosis, the OR for metastatic status was large (3.29 [3.13; 3.46]), but associations with other covariates were not noticeably affected. Documented PS close to diagnosis was associated with poorer 1‐year survival (25% of patients with PS died within 1 year, vs 12% without PS).

**Conclusion:**

PS score documentation was variable and generally low. Improved documentation is required if EHRs are to be used as a source of real‐world data.

## 1 Introduction

Indices of performance status (PS) are used by health professionals to evaluate cancer patients' ability to engage in the activities of daily life, and as eligibility criteria for inclusion in clinical trials [1]. The Karnofsky PS index was introduced in 1948 and ranges from 100 (perfect health) to 10 (moribund) for living patients. The Eastern Cooperative Oncology Group (ECOG) score, ranging from 0 (fully active; able to carry on all pre‐disease performance without restriction) to 4 (completely disabled; totally confined to bed or chair), was introduced by Zubrod and colleagues in 1960 (it has also been called the ‘WHO score’ because of its use in WHO publications). The scales are not the same, but evaluations using ECOG and Karnofsky scores are strongly correlated [2, 3]. The PS is correlated with survival in various types of cancers [4].

Despite their widespread use as thresholds in important clinical decisions, PSs are not systematically documented in patients' electronic health records (EHR). Using rule‐based natural language processing, an American study on patients newly diagnosed with colorectal cancer found that only 51% had any PS documentation in the first 3 months following diagnosis [5]. The study used broad criteria to count PS documentation, with 7% of occurrences being for elements other than numeric scores (e.g., “improved PS”, which mentions the PS but does not quantify it). With similar methods, in another study, 60% of patients had PS information as structured data, and this rose to 73% when adding free‐text analysis [6]. Transformer‐based models have also been used to impute PS in clinical notes, with imputed poor PS associated with worse patient overall survival [7].

The objective of this study was to assess the prevalence of PS documentation in patients newly diagnosed with cancer in public university hospitals in Paris, France, and to analyse clinical factors associated with PS documentation.

## 2 Methods

### 2.1 Study Population

We used the Clinical Data Warehouse (CDW) of Assistance Publique‐Hôpitaux de Paris (Greater Paris university hospital, AP‐HP) to obtain data. AP‐HP is the main university hospital in Paris region. It operates 39 hospitals, and its CDW contains data for 11 million patients [8].

We included all patients aged 18 and above with a new cancer diagnosis at AP‐HP between January 1, 2019, and June 1, 2021, to ensure 365 days of follow‐up for all patients. A ‘new diagnosis’ was defined as the absence of a cancer ICD‐10 code in the previous 24 months. We obtained age at diagnosis, gender, hospital of diagnosis, and metastatic status from claims data. We determined the type of cancer using ICD‐10 claims data and grouped cancers according to primary cancer sites. We categorized patients based on the first ICD‐10 cancer code found in their EHR data; we did not check for possible other cancer codes appearing later. Patients were assigned to the hospital where their first ICD‐10 cancer code was recorded.

We obtained dates of death through linkage with the INSEE national registry of deaths. The last update of these data was on 1 June 2022. We retrieved all available documents in the patients' EHR from AP‐HP's CDW, and kept multidisciplinary team meeting (MDT) reports, consultation reports, and hospitalization reports within −90 and +365 days of the first cancer‐related ICD‐10 code.

We excluded patients who received their first cancer code in hospitals that accounted for less than 1% of diagnoses (mostly long‐term care facilities) and patients whose diagnosis was recorded in paediatric hospitals. We excluded male patients coded with gynaecological cancers (but included male patients with breast cancer), and female patients coded with prostate cancer. We excluded patients without any document in the CDW (i.e., where only claims data were available).

### 2.2 Rule‐Based Approach to Extract PS Scores

PS scores are not available as structured data in AP‐HP's CDW and have to be extracted from free‐text documents. We developed a regular expression (RegEx) to capture the presence of PS scores under their various names (Karnofsky/Zubrod/ECOG/PS/WHO). We combined this RegEx with other rule‐based algorithms from the eds‐nlp open source library for French clinical NLP, which check if a RegEx result applies to a family member (e.g., ‘mother had PS = 3’) or if it is part of a negative statement (e.g., ‘PS is not 0’) [9].

We annotated a first series of documents to develop the rules (110 MDT reports, 75 consultation reports, 75 hospitalization reports, randomly selected), and a second series of documents to assess the performance of the RegEx (70 MDT reports, 70 consultation reports, 100 hospitalization reports, randomly selected). Details are provided in Supporting material.

### 2.3 Statistical Analysis

We conducted descriptive statistical analysis to count the number of documents per patient and per hospital, and the prevalence of PS score documentation between −90 and +365 days of the first cancer code per patient, cancer type, and hospital. We computed Spearman's rho to evaluate the association between the number of documents in a hospital (a proxy for activity in cancer care) and the proportion of those documents that contained a PS score (to assess if larger hospitals document PS more often, which could suggest a learning effect).

We fitted logistic regressions for the presence of a PS score in the patient record, with the following covariates: gender, age at diagnosis (as a restricted cubic spline with 3 knots), cancer type, hospital of diagnosis, and number of each type of reports (MDT, consultation, and hospitalization) available for the patient. We then performed the same analysis with the addition of metastatic status at diagnosis (i.e., within + 90 days of the first ICD‐10 cancer code) as a covariate. We reported odds ratios (ORs) and 95% confidence intervals.

Data processing was conducted using Python, with packages pandas, PySpark for database access and SQL querying, and re for RegEx development. Statistical analysis and visualization was conducted using R (v4.3.1), with packages dplyr, ggplot2 [10], rms [11], patchwork [12].

## 3 Results

### 3.1 Population

We included 68,479 patients who received a first cancer code in 15 different hospitals. Table 1 summarizes the characteristics of these patients and the documents contained in their records. These patients' records contained 649,792 MDT, consultation, and hospitalization reports. 142,768 of these reports (22%) contained a PS score.

**Table 1:** Characteristics of the study population.

		All	With documented PS	Without documented PS
		*N* (col %)	*N* (row %)	*N* (row %)
Total		68.479	28,105 (41%)	40,374 (59%)
Sex	‐ Female	31,869 (47%)	12,972 (41%)	18,897 (59%)
	‐ Male	36,610 (53%)	15,133 (41%)	21,477 (59%)
Cancer type	‐ Breast	5,429 (7.9%)	3,104 (57%)	2,325 (43%)
	‐ Colorectal	5,863 (8.6%)	2,253 (38%)	3,610 (62%)
	‐ Genitourinary	5,359 (7.8%)	1,434 (27%)	3,925 (73%)
	‐ Gynaecological	2,732 (4.0%)	1,158 (42%)	1,574 (58%)
	‐ Haematological	10,883 (16%)	5,276 (48%)	5,607 (52%)
	‐ Lung	5,940 (8.7%)	3,474 (58%)	2,466 (42%)
	‐ Other	11,341 (17%)	3,464 (31%)	7,877 (69%)
	‐ Other gastrointestinal	10,167 (15%)	5,133 (50%)	5,034 (50%)
	‐ Prostate	3,821 (5.6%)	1,294 (34%)	2,527 (66%)
	‐ Skin	6,944 (10%)	1,515 (22%)	5,429 (78%)
Hospital of first cancer‐related ICD‐10 code	‐ Hospital 1	11,195 (16%)	4,572 (41%)	6,623 (59%)
	‐ Hospital 2	3,358 (4.9%)	707 (21%)	2,651 (79%)
	‐ Hospital 3	3,221 (4.7%)	1,332 (41%)	1,889 (59%)
	‐ Hospital 4	3,387 (4.9%)	1,367 (40%)	2,020 (60%)
	‐ Hospital 5	3,515 (5.1%)	657 (19%)	2,858 (81%)
	‐ Hospital 6	2,991 (4.4%)	1,125 (38%)	1,866 (62%)
	‐ Hospital 7	9,537 (14%)	2,746 (29%)	6,791 (71%)
	‐ Hospital 8	6,734 (9.8%)	3,271 (49%)	3,463 (51%)
	‐ Hospital 9	834 (1.2%)	257 (31%)	577 (69%)
	‐ Hospital 10	1,412 (2.1%)	504 (36%)	908 (64%)
	‐ Hospital 11	1,743 (2.5%)	882 (51%)	861 (49%)
	‐ Hospital 12	1,074 (1.6%)	216 (20%)	858 (80%)
	‐ Hospital 13	3,819 (5.6%)	1,592 (42%)	2,227 (58%)
	‐ Hospital 14	10,086 (15%)	6,344 (63%)	3,742 (37%)
	‐ Hospital 15	5,573 (8.1%)	2,533 (45%)	3,040 (55%)
Metastatic at first code		14,971 (22%)	10,039 (67%)	4,932 (33%)
Died within one year		11,697 (17%)	6,967 (60%)	4,730 (40%)

		Median (IQR)	Median (IQR)	Median (IQR)
Number of documents per patient	‐ All	6 (3, 11)	10 (6, 16)	4 (2, 7)
	‐ MDT reports	1 (0, 2)	1 (0, 3)	0 (0, 1)
	‐ Consultation reports	3 (1, 6)	5 (2, 9)	2 (0, 4)
	‐ Hospitalization reports	1 (1, 3)	3 (1, 6)	1 (0, 2)
Age at first cancer‐related ICD‐10 code		66 (55, 75)	67 (56, 75)	66 (55, 76)

Abbreviations: ICD‐10, International Classification of Disease, 10th edition; IQR, InterQuartile Range; MDT, MultiDisciplinary Team meeting.

The number of patients per hospital varied extensively, from 834 to 11,195. The median number of documents per patient was six, but there were 3,221 patients (4.7%) without any document of interest in the EHR. These patients had similar age (66, IQR [54;76]) and gender (53% female) characteristics to the general study population, but were more likely to have skin cancer (28% of patients without documents vs. 9.2% overall). They were also less likely to die within 1 year (8.0% vs. 18%).

### 3.2 Proportion of Documents With a Mention of a Performance Score

Our RegEx performed well in the validation set (accuracy, weighted average F1 score, and macro average F1 score all > 0.95 for all document types, see Supporting materials).

**Figure 1:**
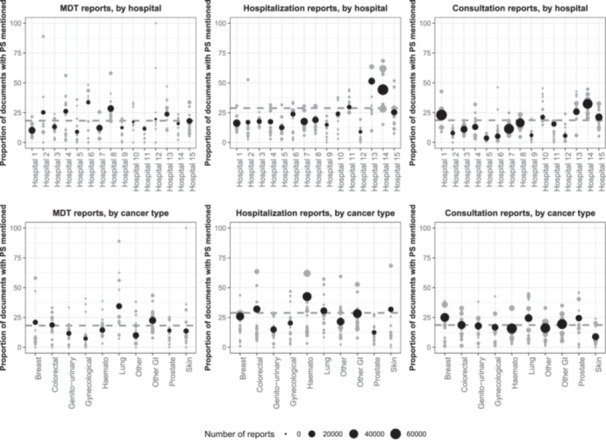
Proportion of documents that contain a PS, for MDT reports (left), hospitalization reports (center), and consultation reports (right), by hospital (top) and by cancer type (bottom). Grey dots represent the proportion for a [hospital * cancer type] combination. Black dots represent the overall proportion for a hospital or a cancer type. The dashed grey line represents the average across hospitals and cancer types. GI, gastroinstestinal.

In the full set of documents, the proportion of patients with a documented PS varied by hospital (19% to 63%) and by type of cancer (22%, skin, to 58%, lung) (Table 1). For any given hospital (cancer type), the prevalence of performance status documentation varied by cancer type (hospital) (Figure 1). On average, PSs were rarely mentioned in reports: 18% of MDT reports contained a PS, 29% of hospitalization reports, and 19% of consultation reports. For hospitalization reports, the proportion rose to 35% when we only considered reports linked to a hospital stay that was coded with a cancer ICD‐10 code. Some combinations of hospital and cancer type had high completion rates in MDT reports, over 75%, but always for small groups of patients.

We analysed the correlation between the number of documents in a hospital and the proportion of documents from this hospital that contained a PS. For hospitalization reports, Spearman's rho = 0.37 (*p* = 0.18) (when restricted to hospitalization reports corresponding to stays coded with a cancer‐related ICD‐10 code, *ρ* = 0.47 with *p* = 0.08); for MDT reports, *ρ* = 0.02 (*p* = 0.94); for consultation reports, *ρ* = 0.58 (*p* = 0.02).

### 3.3 Factors Associated With the Presence of a Score for a Patient

**Figure 2:**
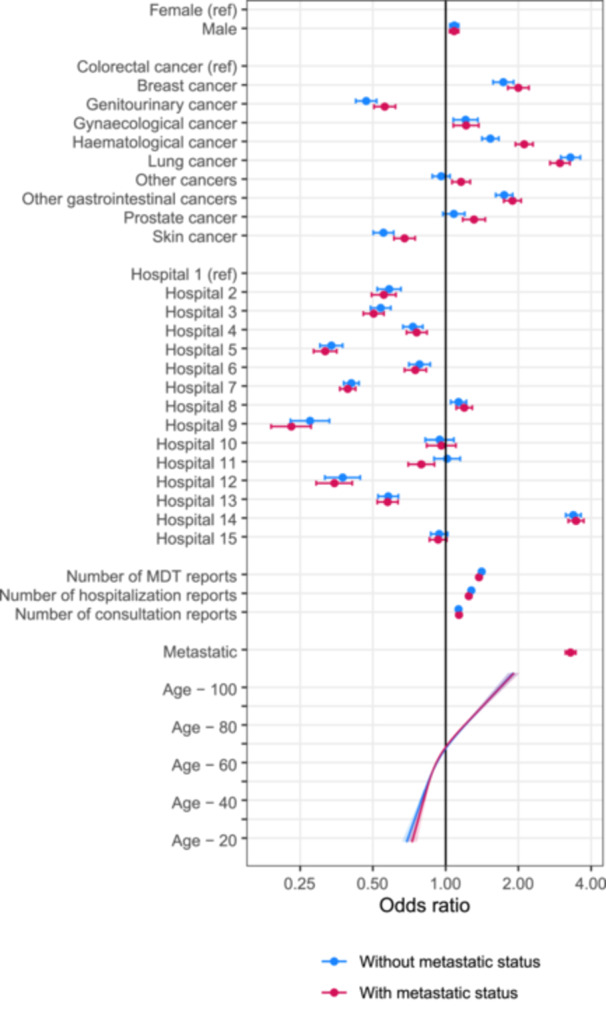
Odds ratio for the presence of a documented PS in the −90 to +360 days around the first cancer‐related ICD‐10 code. Two models were fitted: with, and without integrating information on metastatic status at diagnosis. MDT: MultiDisciplinary Team.

ORs for PS documentation varied extensively by cancer type, from 0.47 (95% confidence interval: [0.42; 0.52]) for genitourinary cancers to 3.30 [3.00; 3.61] for lung cancer, and by hospital, from 0.27 [0.23; 0.33] to 3.38 [3.14; 3.63] (Figure 2). Male patients were slightly more likely to have a documented PS score (OR = 1.08 [1.04;1.13]), and the probability of having an OR documented increased with age. The number of each type of document was associated with the presence of a score: the more documents, the more likely a PS score was recorded in at least one of them.

Adjusting for metastatic status did not noticeably affect associations with other covariates. Patients with recorded metastatic disease were considerably more likely to have a recorded PS (OR 3.29 [3.13; 3.46]).

## 4 Discussion

PS was poorly documented: only 41% of patients had a score. Among the document types with the highest prevalence, reports of hospitalization coded with an ICD‐10 cancer code, only a third of documents (35%) contained a PS score. The low prevalence of PS scores in MDT reports is of concern, since MDT meetings are where cancer treatments are discussed collectively and often should not be provided to patients with a high PS score. Even for a given type of cancer, prevalence varied by hospital. Hospitals with more activity in cancer were more likely to include PS in their hospitalization and consultation reports, but not in their MDT reports.

Male patients, patients with poor‐prognosis cancers (e.g., lung), older patients, and patients who were metastatic at diagnosis were much more likely to have a PS score in their notes, which indicates that physicians document PS scores more for advanced patients. Having a PS score documented around your first cancer ICD‐10 code was also associated with poorer 1‐year survival, suggesting, here again, that PS score documentation is more frequent for patients who are in poorer condition. This, in clinical terms, makes sense, as it shows that clinicians probably tend to discuss the PS when patients deteriorate.

In medical informatics terms, however, having more frequent PS documentation for certain types of patients is a case of informative presence bias, where sicker patients have more data because of more contacts with the health system [13]. This would normally not be the case in a traditional prospective cohort study, where variables are collected systematically. Here, it is likely that PS is missing‐not‐at‐random (i.e., the probability of a missing PS depends on the value of the PS), posing an issue for analysis. Missing PS becomes a confounder.

We can only speculate on why PS documentation varies so much between hospitals, and inside one hospital, between cancer sites. Software could play a role. MDT report templates varied between sites, and some had structured fields for PS. Organizational arrangements could also interfere. Some hospitals had a dedicated secretary for MDTs, while in others, doctors took the notes. Finally, local cultures in certain departments may also place an emphasis on documenting specific variables. Fieldwork in each department would be needed to understand how documentation is done ‘at the coalface’.

The prevalence of PS score documentation we found is much lower than in the study by Agaronnik et al. (51%). However, they had more selective inclusion criteria, with patients between 21 and 75 years old, a focus on colorectal cancer, and a requirement to have at least a second colorectal cancer code between 30 and 365 days after the initial code. Cohen et al. reached 73% prevalence, but their dataset comprises specific cancer types, e.g., a focus on advanced gastric and pancreatic cancers (which have a poor prognosis). We did not exclude any patient based on stage. Besides, Cohen et al. obtained most of their scores from structured data, suggesting that structured data collection may improve prevalence (but mandatory structured fields may increase data input time and clinicians' frustration with EHRs [14]). Compared to these two studies, our results reflect current practice for multiple cancer types, regardless of stage or age, in a large, multisite hospital system, with different documentation cultures between departments and no structured field for PS documentation in most documents. In England, the National Lung Cancer Audit found 86% documentation of PS (below the national target of 90%) [15]. It shows that better documentation practices are achievable, after sustained effort (the audit has been conducted annually since 2005, at which point PS documentation was just above 50% [16]).

Importantly, the absence of PS documentation does not mean that there was no clinical assessment of PS. Doctors may well have assessed the patient's general state, and then have forgotten to write it down, considered it of little relevance, or reported on the patient's state in an unstructured fashion (such as ‘patient in good overall condition’). Therefore, lack of documentation does not directly reflect clinical practice.

### 4.1 Limitations

Our study has limits. We only cover AP‐HP, a large, public, university hospital, and it is not clear to what extent the results can be generalized to non‐university hospitals, private hospitals, or cancer treatment centres. We rely entirely on document availability, and therefore on the quality of document uploading in AP‐HP's CDW. If data flows are flawed, we may lack documents in the database even if they are present in the initial hospital software [17]. Here, 4.7% of patients had no eligible document in their record. This might be due to upload issues into the CDW.

We categorised patients based on the first ICD‐10 cancer code that appeared in their record. ICD‐10 coding is used for billing and generally considered reliable, but misclassifications might have happened. For PS, our analysis is limited by the performance of the regular expressions used for extracting PS. We did not perform manual verification (aside from the annotation used to develop and validate the regular expression). We also did not assess if PS documentation was associated with clinical decision‐making (e.g., treatment decisions). There is potentially confounding between healthcare utilization and the likelihood of PS documentation.

## 5 Conclusion

PS is an important information for the evaluation of cancer patients, yet it remains poorly documented in clinical notes. Collecting PS as a structured variable, e.g., in MDT reports, may help improve data completion. Yet, structured documentation must be carefully weighed against the additional workload it would represent—a classic usability concern in EHRs.

## Author Contributions

**Guillaume Lame:** conceptualization, investigation, funding acquisition, writing – original draft, methodology, visualization, writing – review and editing, software, formal analysis. **Mohamed El Mejdani:** investigation, methodology, writing – review and editing, software, formal analysis. **Ariel Cohen:** methodology, writing – review and editing, software, formal analysis. **Sonia Priou:** methodology, writing ‐ review and editing, software, formal analysis. **Remi Flicoteaux:** conceptualization, methodology, writing – review and editing, software, formal analysis. **Matthew Barclay:** visualization, writing – review and editing, software, formal analysis. **Christophe Tournigand:** conceptualization, methodology, writing – review and editing, formal analysis. **Marie Verdoux:** methodology, writing – review and editing, software, formal analysis. **Emmanuelle Kempf:** conceptualization, investigation, methodology, writing – review and editing, formal analysis.

## Ethics statement

This study was approved by the APHP's Scientific and Ethics Committee (IRB00011591; approval CSE 20‐0055_COVONCO‐AP) on 15 May 2020.

## Conflicts of Interest

The authors declare no conflicts of interest.

## Supporting information

Performance status appendix.

## Data Availability

The data that support the findings of this study are available from Assistance Publique ‐ Hôpitaux de Paris. Restrictions apply to the availability of these data, which were used under license for this study. Data are available from https://eds.aphp.fr/ with the permission of Assistance Publique ‐ Hôpitaux de Paris.
