# Supplementary material for: A Comprehensive Assessment of Cancer Patient Performance Status Documentation in a Large, Multicentre Hospital System
Source: J Eval Clin Pract. 2026 Mar 15;32(2):e70411. doi: 10.1111/jep.70411 (PMC12989242; doi:10.1111/jep.70411)
Supplement: Supplementary file 1 — Performance status appendix. [file JEP-32-0-s001.pdf]

Appendix - RegEx development and validation analysis

We annotated 110 MDT reports, 75 hospitalization reports, and 75 consultation reports drawn at random from patients with a cancer code to develop the regular expression.

The objective was to assess the presence of the score, not to extract its value. This second objective is slightly more complicated, because it requires arbitrating between different options if more than one score is available in the document. Besides, we are interested in the determinants of PS score documentation, not in the prognostic value of PS score levels themselves, so knowing whether a score was documented is enough for our purpose.

We settled on the following expression:

```
\b(((oms|ecog|performance status|zubrod)[^\n\d]{0,10}([0-5]{1}(\s|\p{P})))|((ps)([^al0-9\n]{0,10})([0-5]{1}(\s|\p{P})))|((ip)([^pis0-9\n]{0,10})([0-5]{1}(\s|\p{P})))|((karnofsky|ik)[^\n]{0,10}((0|100|[1-9][0-9])))|(d[eéè]c[éeè][sd].{0,4}))|^(?!.*\b(COVID|SARS)\b).*asymptomatique'
```

Table S1 explains the construction of this RegEx.

Table S1. Explanation of the regular expression.

<table>
<tr><th></th><th>RegEx</th><th>Explanation</th></tr>
<tr><td>1</td><td>'\b</td><td>New word</td></tr>
<tr><td>2</td><td>(((oms|ecog|performance status|zubrod)[^\n\d]{0,10}([0-5]{1}(\s|\p{P})))|</td><td>OMS or ECOG or ‘performance status’ or Zubrod, followed by 0 to 10 characters that are neither digits nor a new line, followed by a number between 0 and 5, followed by any punctuation OR</td></tr>
<tr><td>3</td><td>((ps)([^al0-9\n]{0,10})([0-5]{1}(\s|\p{P})))|</td><td>‘ps’ followed by 0 to 10 characters that are neither a nor l nor a digit (so no PSA or PSL), followed by a number between 0 and 5, followed by any punctuation OR</td></tr>
<tr><td>4</td><td>((ip)([^pis0-9\n]{0,10})([0-5]{1}(\s|\p{P})))|</td><td>‘ip’ followed by 0 to 10 characters that are neither p nor I nor s nor a digit (so no IPP, IPI or IPS), followed by a number between 0 and 5, followed by any punctuation OR</td></tr>
<tr><td>5</td><td>((karnofsky|ik)[^\n]{0,10}((0|100|[1-9][0-9])))|</td><td>‘karnofsky’ or ‘ik’ followed by 0 to 10 characters except a new line, followed by 0, 100 or any figure between 1 and 99 OR</td></tr>
<tr><td>6</td><td>(d[eéè]c[éeè][sd].{0,4}))|</td><td>‘deces’ or ‘deced’ (with or without any combination of accents) followed by 0 to 4 of any character OR (death is ECOG=5)</td></tr>
<tr><td>7</td><td>^(?!.*\b(COVID|SARS)\b).*asymptomatique'</td><td>at the start of a line, look ahead to check that the words “COVID’ or ‘SARS’ are not present, then check if ‘asymptomatique’ is present (‘asymptomatique’ = asymptomatic, was equated as ECOG=0)</td></tr>
</table>

We made the search case-insensitive, and we combined this RegEx with pre-existing pipelines from the 'eds-nlp' package developed by AP-HP's Data Science team to ensure that the regex did not apply to another family member, and that it was not a negation.[9]

We then annotated another 70 MDT reports, 100 hospitalization reports and 70 consultation reports for performance assessment.

We computed the macro average and weighted average F1 score and the accuracy. Weighted averages are combined using weights proportional to the prevalence of positive and negative records in the dataset, whereas macro averages are averages with weights of 0.5 for positive and negative records; therefore, they are equal if the dataset has 50% positive records but their values can differ a lot if the dataset is unbalanced towards positive or negative records.

The performance we obtained on the development and validation sets are detailed in Table S2.

Table S2. RegEx performance on train and test sets. Avg: average, acc: accuracy.

Set	N false	N true	Doc type	Acc.	Macro avg F1	Weighted avg F1
Development	91	19	MDT reports	0.94	0.90	0.94
Development	37	38	Hospitalization reports	0.97	0.97	0.97
Development	59	16	Consultation reports	0.97	0.96	0.97
Validation	57	13	MDT reports	0.97	0.96	0.97
Validation	46	52	Hospitalization reports	0.98	0.98	0.98
Validation	55	15	Consultation reports	0.97	0.96	0.97

This regular expression was developed for general use in AP-HP's CDW. However, in practice, people do not normally talk about a patient being 'PS 5', i.e., deceased.

For the present work, we omitted section 6 (Table S1) from the RegEx to avoid biasing the results with mentions of decease and rather focus on performance status evaluation prior to decease. In total, we started with 671,635 documents, of which 21,490 contained at least one hit for section 6 of the RegEx, and 145,479 contained one hit for the RegEx without section 6.

Full results on development set

MDT reports

annotation_OMS	False	True
regex_output		
False	84	7
True	0	19

Hospitalization reports

annotation_OMS	False	True
regex_output		
False	37	0
True	2	36

Consultation reports

annotation_OMS	False	True
regex_output		
False	57	2
True	0	16

Full results on validation set

MDT reports

annotation_OMS	False	True
regex_output		
False	55	2
True	0	13

Hospitalization reports

annotation_OMS	False	True
regex_output		
False	46	0
True	2	50

Consultation reports

annotation_OMS	False	True
regex_output		
False	54	1
True	1	14
